# CT-Based Radiomics Nomogram Improves Risk Stratification and Prediction of Early Recurrence in Hepatocellular Carcinoma After Partial Hepatectomy

**DOI:** 10.3389/fonc.2022.896002

**Published:** 2022-07-07

**Authors:** Cuiyun Wu, Shufeng Yu, Yang Zhang, Li Zhu, Shuangxi Chen, Yang Liu

**Affiliations:** ^1^ Cancer Center, Department of Radiology, Zhejiang Provincial People’s Hospital (Affiliated People’s Hospital, Hangzhou Medical College), Hangzhou, China; ^2^ Cancer Center, Department of Ultrasound Medicine, Zhejiang Provincial People’s Hospital (Affiliated People’s Hospital, Hangzhou Medical College), Hangzhou, China; ^3^ Key Laboratory of Gastroenterology of Zhejiang Province, Zhejiang Provincial People's Hospital (Affiliated People's Hospital, Hangzhou Medical College), Hangzhou, China

**Keywords:** hepatocellular carcinoma, recurrence, radiomics, machine learning, models, nomograms

## Abstract

**Objectives:**

To develop and validate an intuitive computed tomography (CT)-based radiomics nomogram for the prediction and risk stratification of early recurrence (ER) in hepatocellular carcinoma (HCC) patients after partial hepatectomy.

**Methods:**

A total of 132 HCC patients treated with partial hepatectomy were retrospectively enrolled and assigned to training and test sets. Least absolute shrinkage and selection operator and gradient boosting decision tree were used to extract quantitative radiomics features from preoperative contrast-enhanced CT images of the HCC patients. The radiomics features with predictive value for ER were used, either alone or in combination with other predictive features, to construct predictive models. The best performing model was then selected to develop an intuitive, simple-to-use nomogram, and its performance in the prediction and risk stratification of ER was evaluated using the area under the receiver operating characteristic curve (AUC), calibration curve, and decision curve analysis (DCA).

**Results:**

The radiomics model based on the radiomics score (Rad-score) achieved AUCs of 0.870 and 0.890 in the training and test sets, respectively. Among the six predictive models, the combined model based on the Rad-score, Edmondson grade, and tumor size had the highest AUCs of 0.907 in the training set and 0.948 in the test set and was used to develop an intuitive nomogram. Notably, the calibration curve and DCA for the nomogram showed good calibration and clinical application. Moreover, the risk of ER was significantly different between the high- and low-risk groups stratified by the nomogram (*p <*0.001).

**Conclusions:**

The CT-based radiomics nomogram developed in this study exhibits outstanding performance for ER prediction and risk stratification. As such, this intuitive nomogram holds promise as a more effective and user-friendly tool in predicting ER for HCC patients after partial hepatectomy.

## Introduction

Hepatocellular carcinoma (HCC), a predominant form of liver cancer (1, 2), is the sixth most common cancer globally and the fourth highest cause of cancer-related deaths (3). At present, surgical resection is the main treatment for HCC, but the recurrence rate remains very high in HCC patients after surgery. It has been shown that recurrence usually occurs in approximately 70% of patients within 1-3 years after surgery (4). Additionally, the prognosis of early recurrence (ER) (≤1 year following surgery) was significantly worse than that of late recurrence (>1 year) (5, 6). Moreover, tumor recurrence is the primary cause of poor survival in HCC patients (7) as well as the leading cause of death in the first year (6). Therefore, it is urgent to identify effective predictors of ER that can be used for the risk stratification of HCC patients to guide treatment decisions.

Previous studies (8–11) identified a number of factors associated with tumor recurrence, such as serum alpha-fetoprotein (AFP), microvascular invasion (MVI), pathological grade, venous invasion, and tumor size, but the results were inconsistent among the studies. In addition, these recurrence-related factors have limitations. For instance, pathological features can only be obtained using invasive biopsy techniques. Due to the large spatial and temporal heterogeneity of solid cancers (12), features identified through a biopsy cannot fully reflect the features of the entire lesion. In recent years, radiomics has emerged as a non-invasive, high-throughput imaging technology that enables the extraction of large amounts of imaging features from medical images, transforming the qualitative analysis of traditional images to quantitative analysis. Radiomics has great potential to capture heterogeneity within tumors in a non-invasive manner (12) by utilizing high-throughput information hidden in images to provide information on tumor pathophysiology (13, 14) and contribute to precision medicine (15, 16). The technique has been widely applied in the diagnosis, treatment, and prognosis of many types of cancer (e.g. head and neck cancer, colorectal cancer, breast cancer, glioma, cervical cancer) (17–21). Radiomics can reflect the heterogeneity of tumors (22), evaluate the biological behavior of tumors as a whole, and provide support for clinical decision-making.

Currently, HCC staging systems such as the Barcelona Clinical Liver Cancer (BCLC), tumor lymph node metastasis, and Cancer of the Liver Italian Program systems play an important role in prognostic prediction (23). However, these systems have limitations in predicting early postoperative recurrence and fail to quantify risk measures (24). Some previous studies applied radiomics to supplement existing staging systems to compensate for the shortcomings of existing models. At present, several studies have been conducted to predict the recurrence of HCC using computed tomography (CT) radiomics (24–29). However, only a few reports have predicted ER in HCC patients using CT radiomics. These studies found that radiomics had better semantic feature performance than traditional images. In addition, there are few reports on nomogram-based radiomics to predict risk stratification in high- and low-risk groups.

This study aimed to construct and validate a CT-based radiomics nomogram for predicting postoperative ER in HCC patients. The findings gained through conducting this study may provide clinicians with a novel intuitive nomogram as a better approach for individual risk prediction of ER, eventually improving the survival rate for HCC patients following liver resection.

## Materials and Methods

### Patients

**Figure 1:**
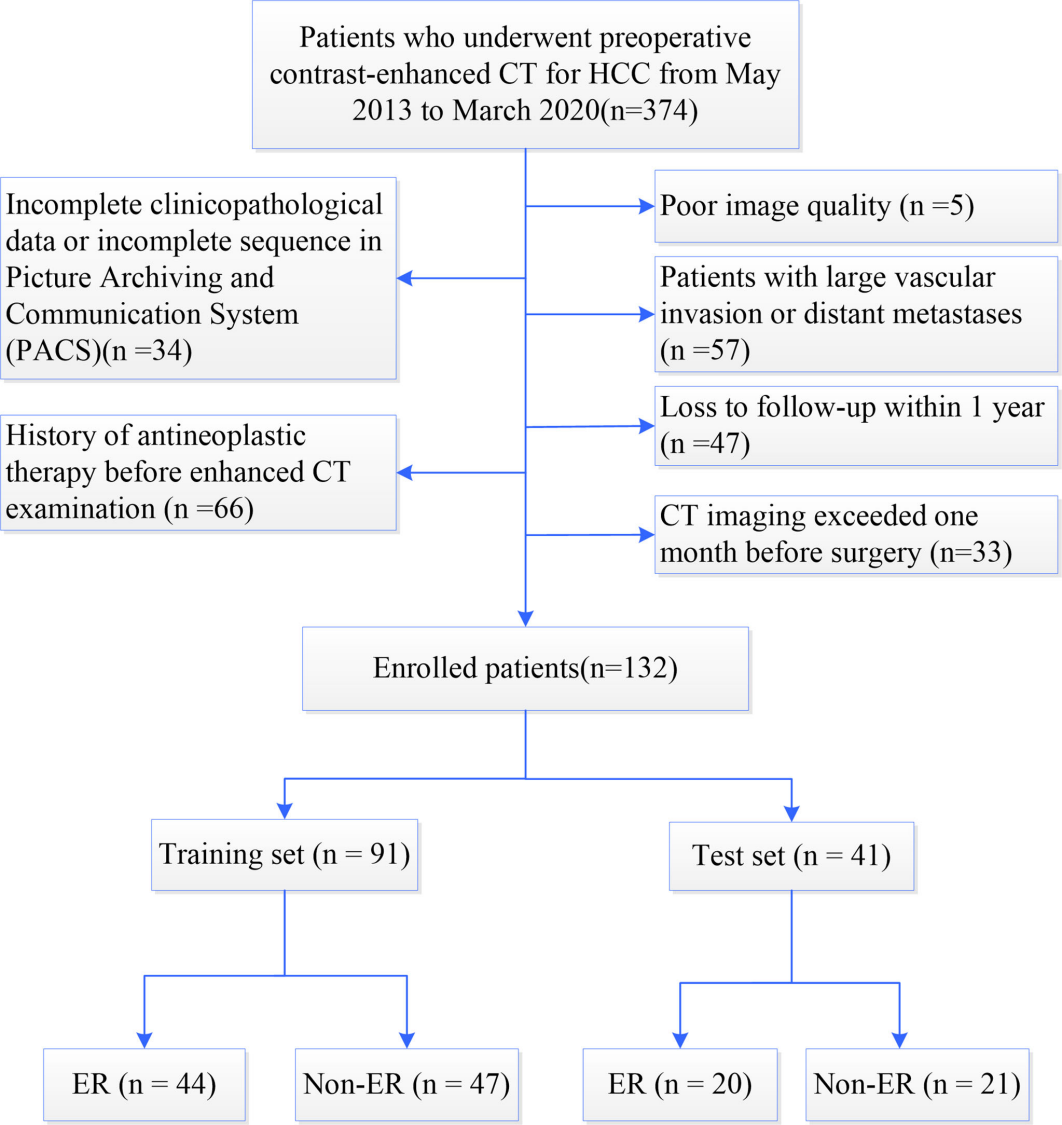
Flowchart of patient enrollment. HCC, hepatocellular carcinoma; CT, computed tomography; ER, early recurrence; Non-ER, non-early recurrence.

A total of 374 HCC patients who underwent preoperative contrast-enhanced CT during the period spanning nearly 7 years from May 2013 to March 2020 in our hospital were screened to determine their eligibility. The inclusion and exclusion criteria during patient enrollment were described in **Supplementary Methods**. After excluding 242 ineligible patients, 132 patients were retrospectively enrolled in this study (**Figure 1**). According to the time of CT examination, the included HCC patients were assigned to the following two groups at a ratio of 7:3: the training set (n=91) used to construct the model and the test set (n=41) used to assess the performance and verify the reliability of the model.

The Institutional Ethics Committee approved this retrospective study and waived written informed consent owing to the retrospective nature of the study (Approval No. 2019KY216).

### Data Collection, CT Imaging Procedure, and Follow-Up

Demographic characteristics and clinical information, including age, sex, AFP, BCLC staging, MVI, Edmondson grade, etc., were collected for each patient. Preoperative CT images for each HCC patient were reviewed and evaluated independently by two experienced radiologists who determined the characteristics of the tumor (e.g. number, size) and other features (e.g. liver cirrhosis, tumor capsule). In the event of disagreements, the CT images were reassessed until an agreement was reached. Detailed information about the data collection and CT imaging procedure was presented in the **Supplementary Methods**. Enhanced CT image acquisition and scanning parameters were provided in the **Supplementary Methods** and **Supplementary Table S1**.

Follow-up examinations, including laboratory tests (e.g. serum AFP levels, liver function tests) and imaging examinations [ultrasound, CT, or magnetic resonance imaging (MRI)] were performed every three months after surgery. The primary endpoint was tumor ER, which was defined as new intrahepatic or extrahepatic tumors occurring within one year after treatment and determined based on the observation of typical imaging features or pathological confirmation of HCC. The definition of recurrence-free survival (RFS) was a one-year period from the date of surgery to the date of first recurrence or metastasis (radiologically or histologically confirmed).

### Image Preprocessing, Segmentation, and Feature Extraction

Each CT image was standardized using GE AK software (Analysis Kit). The images were resampled with a resolution of 1 x 1 x 1 mm^3^, denoised, grey-normalized, and discretized to order 32. ITK-SNAP software (http://www.itksnap.org/) was used to manually segment the entire HCC layer by layer and delineate the region of interest along the edge of the lesion to obtain a three-dimensional volume of interest (VOI). The CT images were evaluated independently by two radiologists who were blinded to the study, Radiologist 1 and Radiologist 2 delineated the VOIs of the same 30 random images respectively. One week later, Radiologist 1 delineated the VOIs of the same 30 images again. Finally, Radiologist 1 completed the remaining VOIs. The repeatability of feature extraction between inter- and intra-observer was evaluated with the intraclass correlation coefficient (ICC). Feature selection with ICC higher than 0.80 resulted in satisfactory repeatability and reliability.

The VOIs of each sequence were imported into AK software and 396 radiomics features were extracted including the histogram, form factor, gray-level co-occurrence matrix (GLCM), Haralick features, run-length matrix (RLM), and gray-level size zone matrix (GLSZM). All radiomics features were illustrated in **Supplementary Figure S1**. Seven hundred ninety-two features were obtained from each patient during the arterial phase (AP) and the portal venous phase (PP) scans of the abdomen.

### Identification of the Radiomics Signature

Dimension reduction using the training set was performed using analysis of variance (ANOVA), correlation analysis, and least absolute shrinkage and selection operator (LASSO). Subsequently, the new gradient boosting decision tree algorithm was adopted to further reduce the number of dimensions. The concrete steps were shown in the **Supplementary details of dimension reduction,** **Supplementary Table S2**, and **Supplementary Figure S2**. Finally, robust radiomics features were obtained after the completion of logistic regression analysis and five-fold cross-validation. The remaining features were used to establish the radiomics signature. A radiomics score (Rad-score) was assigned to each patient in the training and test sets, calculated using a formula provided in the **Supplementary Methods**.

### Construction of Models and Nomogram, and Performance Evaluation

Variables with a predictive value in the training set were analyzed by univariate analysis, including the above-mentioned clinical characteristics, radiological features, and Rad-score. Variables with *p*<0.1 were included in the multivariate logistic regression analysis. We adopted stepwise backward selection based on the stop rule for the Akaike Information Criterion (AIC) to determine the independent predictors associated with ER, and variables with *p*<0.05 were used to create various models. We used four machine learning methods to determine the performance of the predicted models, namely the logistic regression, Bayes, support vector machine (SVM), and K-nearest neighbor (KNN) algorithms. The diagnostic accuracy of the models was evaluated using the receiver operating characteristic (ROC) curve, and the DeLong test was used to select the best machine learning model. The best-performing machine learning method was used to create the final models.

We constructed a total of six models on the basis of multi-modality approaches: 1) clinical model, 2) radiological model, 3) radiomics model, 4) radiomics-clinical model, 5) radiomics-radiological model, and 6) combined model to determine whether radiomics features could improve the prediction ability. Finally, we used the combined model to develop a nomogram as a visual tool to predict the ER probability of HCC patients.

**Figure 2:**
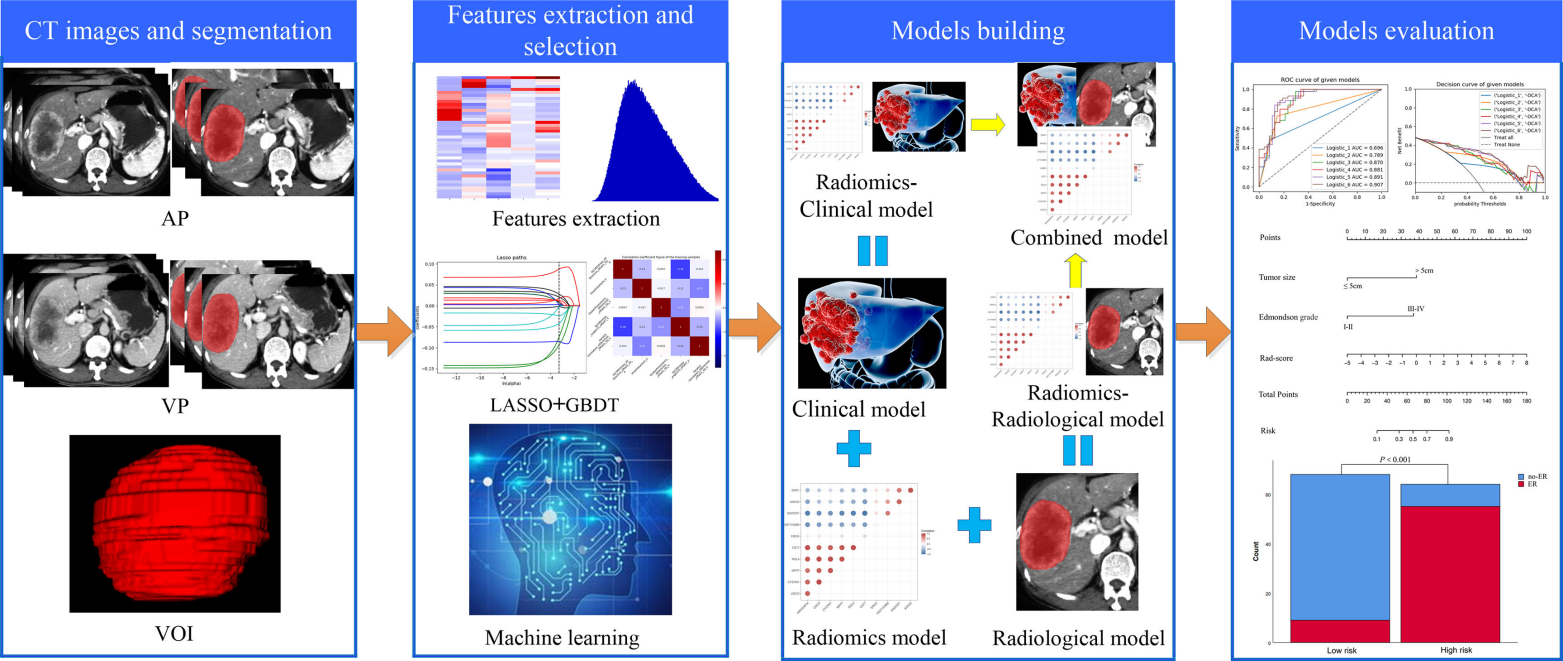
Schematic diagram of construction and evaluation of models.

The ROC curve and decision curve analysis (DCA) were used to evaluate the prediction performance of the models. The area under the ROC curve (AUC), calibration curve, and DCA were used to assess the performance of the nomogram. The ROC curves showed the performance of the six models and the DeLong test was used to compare the differences. The nomogram was constructed based on the combined model to calculate the ER probability for each HCC patient, and the goodness of fit of the nomogram was analyzed using the Hosmer-Lemeshow test. *p*>0.05 was considered to indicate a good test effect. The calibration curve was used to visually evaluate how much the predicted probability of ER offset the actual probability. Then, the optimal cut-off value was calculated from the Youden index (according to ROC curve). The patients were classified into high- and low-risk groups according to the nomogram. The flowchart of model construction and performance evaluation was shown in **Figure 2**.

### Statistical Analysis

Statistical analysis was conducted with SPSS software (version 25.0) and R software (version 3.4). The Shapiro-Wilk test was used to test for the normal distribution of continuous variables. Normally distributed continuous variables were expressed as the mean ± standard deviation (SD) while the non-normal variables were presented as the median (upper and lower quartiles). The independent-sample t test or Mann-Whitney U test was used for continuous variables, while the Chi-squared test or Fisher’s exact test was used for categorical variables, which were expressed as numbers (percentages). The goodness of fit was evaluated with the Hosmer-Lemeshow test. ROC curves were created using MedCalc software (version 19.6), and the DeLong test was used to compare the difference in AUCs between groups. Calibration curves and nomograms were constructed using the “rms” package in R software, and DCA was conducted using the R software package “dca. R”. All statistical tests were two-sided, and *p*<0.05 indicated statistical difference.

## Results

### Demographic and Clinical Characteristics of HCC Patients in the Training and Test Sets

**Figure 3:**
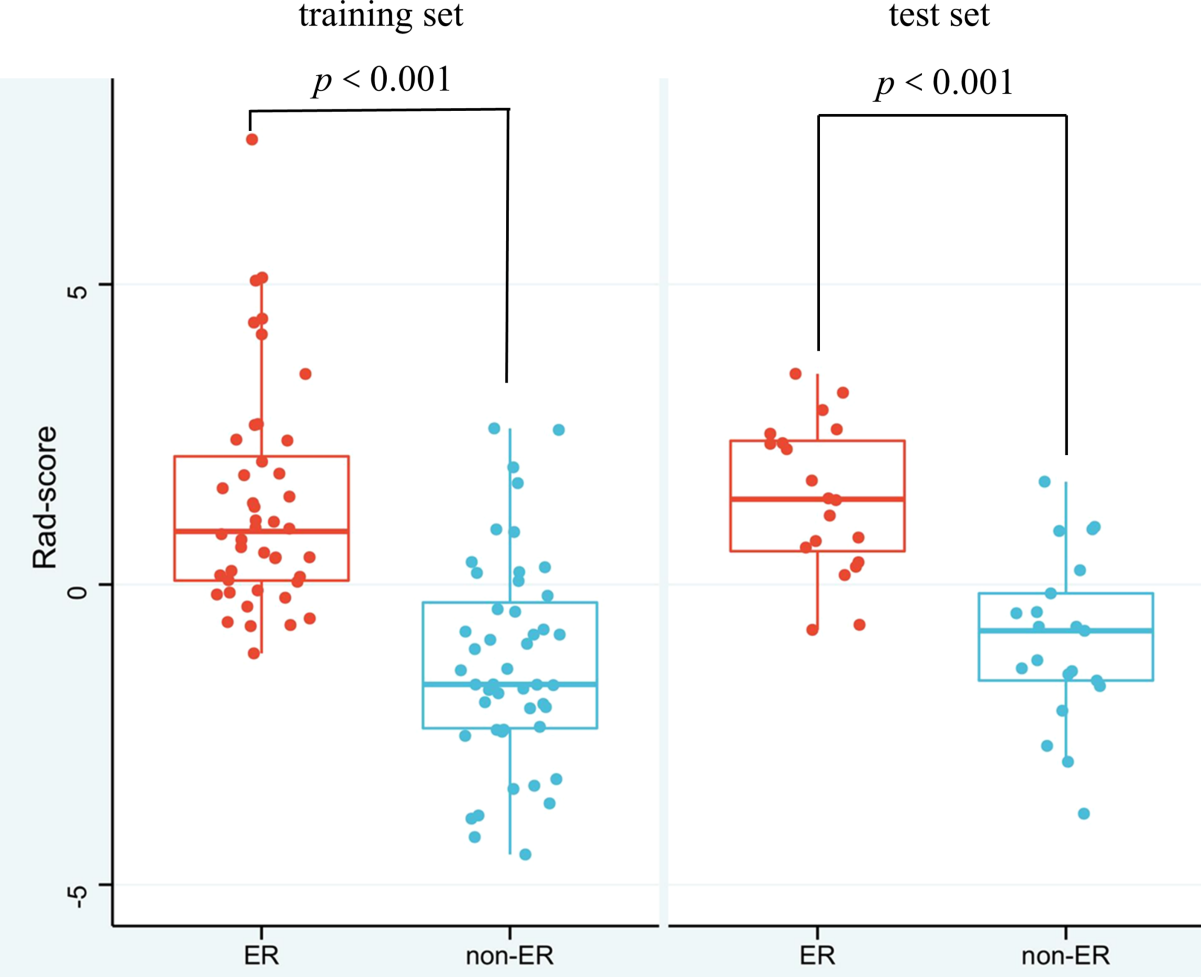
Box-Scatter plots showing that the Rad-score of the ER group is substantially higher than that of the non-ER group both in the training and test sets. Red represents a high Rad-score in the ER group, and blue represents a low Rad-score in the non-ER group, indicating that the higher the Rad-score is, the more likely ER is in HCC patients (ER, early recurrence; non-ER, non-early recurrence).

The enrolled 132 patients were divided into training (n = 91) and test (n = 41) sets at a ratio of 7:3 and subdivided into ER and non-ER groups. The demographic characteristics, laboratory tests, and clinical features were summarized in **Table 1**, and the radiologic features of HCC were listed in **Table 2**. There were no significant differences in these characteristics between the training and test sets (all *p*>0.05). In terms of the ER and non-ER groups, univariate analysis revealed significant differences in lnAFP (AFP was processed by natural logarithm), MVI, Edmondson grade, tumor size, capsule appearance, arterial phase (AP) peritumoral enhancement, and intratumoral necrosis between the two groups in the training and test sets (all *p*<0.05). In addition, the AST and BCLC stages for the training set and the Child-Pugh grade and tumor margins for the test set were statistically different between the ER and non-ER groups (all *p*<0.05). These variables were potential predictors of ER in HCC patients. In the training and test sets, the Rad-scores of the ER and non-ER groups were statistically different (*p*<0.001) (**Table 2**). It was of note that the Rad-score of ER patients was higher than that of non-ER patients (**Figure 3**, **Supplementary Figure S3**).

**Table 1:** Demographic and clinical characteristics of HCC patients in the training and test sets.

Variables	Training set (n = 91)			Test set (n = 41)			*p inter*
	ER(n = 44)	non-ER(n = 47)	*p intra*	ER(n = 20)	non-ER(n = 21)	*p intra*	
Age (years), mean ± SD	56.89 ± 11.45	60.04 ± 9.99	0.164	56.35 ± 11.16	58.05 ± 1.39	0.627	0.526
Gender, no. (%)			0.425			0.606	0.5
Female	4 (9.1)	2 (4.3)		1 (5.0)	3 (14.3)		
Male	40 (90.9)	45 (95.7)		19 (95.0)	18 (85.7)		
HBs-Ag, no. (%)			0.734			1	0.997
Negative	9 (20.5)	11 (23.4)		4 (20.0)	5 (23.8)		
Positive	35 (79.5)	36 (76.7)		16 (80.0)	16 (76.2)		
ALT (U/L), no. (%)			0.147			0.939	0.857
≤50	32 (72.7)	40 (85.1)		16 (80.0)	17 (81.0)		
>50	12 (27.3)	7 (14.9)		4 (20.0)	4 (19.0)		
AST (U/L), no. (%)			0.028*			0.279	0.569
≤40	23 (52.3)	35 (74.5)		10 (50.0)	14 (66.7)		
>40	21 (47.7)	12 (25.5)		10 (50.0)	7 (33.3)		
lnAFP, median (p25, p75)	4.78 (1.93-7.41)	3.23 (1.33-6.42)	0.048*	5.35 (3.58-7.76)	2.10 (1.01-5.40)	0.004*	0.319
Child-Pugh grade, no. (%)			0.425			0.021*	0.316
A	40 (90.9)	45 (95.7)		15 (75.0)	21 (100.0)		
B	4 (9.1)	2 (4.3)		5 (25.0)	0 (0)		
BCLC stage			0.046*			0.606	0.754
0+ A	36 (81.8)	45 (95.7)		18 (90)	20 (95.2)		
B	8 (18.2)	2 (4.3)		2 (10)	1 (4.8)		
MVI, no. (%)			<0.001*			0.003*	0.372
Negative	14 (31.8)	38 (80.9)		5 (25.0)	15 (71.4)		
Positive	30 (68.2)	9 (19.1)		15 (75.0)	6 (28.6)		
Edmondson grade, no. (%)			<0.001*			0.004*	0.71
I-II	23 (52.3)	43 (91.5)		11 (55.0)	20 (95.2)		
III-IV	21 (47.7)	4 (8.5)		9 (45.0)	1 (4.8)		

ER, early recurrence; non-ER, non-early recurrence; HBs-Ag, hepatitis B surface antigen; ALT, alanine aminotransferase; AST, aspartate aminotransferase; ln, natural logarithm; AFP, alpha-fetoprotein; BCLC, Barcelona Clinical Liver Cancer staging, MVI, microvascular invasion; p25 and p75 were the lower and upper quartiles. p intra indicates whether there was a statistical difference between the ER and non-ER groups; p inter indicates whether there was a statistical difference between the training and test sets. *p < 0.05.

**Table 2:** Radiological features and Rad-score of HCC patients in the training and test sets.

Variables	Training set (n = 91)			Test set (n = 41)			*p inter*
	ER(n = 44)	Non-ER(n = 47)	*p* intra	ER(n = 20)	Non-ER(n = 21)	*p intra*	
Tumor number, no. (%)			0.173			1	0.063
1	36 (81.8)	43 (91.5)		20 (100.0)	20 (95.2)		
≥2	8 (18.2)	4 (8.5)		0 (0)	1 (4.8)		
Tumor size, no. (%)			<0.001*			<0.001*	0.709
≤5cm	12 (27.3)	40 (85.1)		5 (25.0)	17 (81.0)		
>5cm	32 (72.7)	7 (14.9)		15 (75.0)	4 (19.0)		
Liver cirrhosis, no. (%)			0.483			0.141	0.11
Absent	23 (52.3)	28 (59.6)		12 (60.0)	17 (81.0)		
Present	21 (47.7)	19 (40.4)		8 (40.0)	4 (19.0)		
Tumor capsule, no. (%)			0.001*			<0.001*	0.745
Complete	19 (43.2)	36 (76.6)		7 (35.0)	19 (90.5)		
Incomplete	25 (56.8)	11 (23.4)		13 (65.0)	2 (9.5)		
Tumor margin, no. (%)			0.078			0.001*	0.36
Smooth	20 (45.5)	30 (63.8)		4 (20.0)	15 (71.4)		
Non-smooth	24 (54.5)	17 (36.2)		16 (80.0)	6 (28.6)		
AP PE, no. (%)			0.019*			0.009*	0.674
Absent	32 (72.7)	43 (91.5)		14 (70.0)	21 (100.0)		
Present	12 (27.3)	4 (8.5)		6 (30.0)	0 (0)		
Intratumoral necrosis, no. (%)			<0.001*			<0.001*	0.344
Absent	7 (15.9)	25 (53.2)		0 (0)	11 (52.4)		
Present	37 (84.1)	22 (46.8)		20 (100)	10 (47.6)		
Rad-score, median (*p*25, *p*75)	0.88 (0.53,2.31)	-1.66 (-2.42, -0.19)	<0.001*	1.42 (0.43, 2.47)	-0.77 (-1.65,0.45)	<0.001*	0.521

ER, early recurrence; non-ER, non-early recurrence; AP, arterial phase; PE, peritumoral enhancement; p25 and p75 were the lower and upper quartiles. *p < 0.05.

### Construction and Performance Evaluation of Models

There were 792 CT-based radiomics features in the AP and PP scans of the abdomen per patient in total. After reducing the dimensions of the feature set, five features with the most predictive value were identified, with one feature in the AP scan and four features in the PP scan of the abdomen. These features were selected for construction of the CT-based radiomics model (**Supplementary Table S3**).

**Figure 4:**
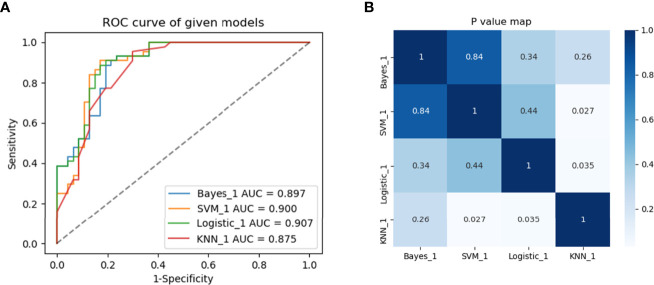
Predictive performance of different machine learning methods. **(A)** Four machine learning methods and **(B)** heat maps comparing *p*-values of AUCs in the training set between groups.

Multivariate logistic regression analysis showed that the Edmondson grade (*p*= 0.028), tumor size (*p*=0.018), and Rad-score (*p*=0.041) were independent predictors of ER (**Table 3**). These three variables were used to establish the combined model. It was worth noting that the combined model had the highest diagnostic performance (AUC = 0.907) among the four machine learning methods in the training set, and the AUCs for the Bayes, SVM, and KNN algorithms were 0.897, 0.900, and 0.875, respectively. The DeLong test showed that the AUC value for logistic regression was not statistically different from that of the Bayes and SVM machine learning methods (*p*>0.05) and was superior to that of the KNN machine learning method (*p*<0.05) (**Figures 4A, B**). Therefore, the logistic regression method was used to develop the models.

**Table 3:** Multivariate logistic regression analysis of the Rad-score and clinical-radiological factors for ER in the training set.

Variables	OR	95% CI	*p*-value
Rad-score	1.705	1.023-2.841	0.041^*^
Edmondson grade	6.614	1.224-35.739	0.028^*^
BCLC stage	5.456	0.701-42.434	0.105
MVI	3.221	0.862-12.038	0.082
Tumor size	6.979	1.399-34.805	0.018^*^

Significant variables with p < 0.1 in the univariate analysis (**Tables 1** & **2**) in the training set were included in the multivariate logistic regression analysis. ER, early recurrence; OR, odds ratio; CI, confidence interval. *p < 0.05.

We used logistic regression machine learning to construct six different prediction models with three independent predictors: Edmondson grade, tumor size, and Rad-score (**Table 4**). The clinical model was established using the Edmondson grade. The radiological model was only based on the tumor size, while the radiomics model was constructed on the basis of the Rad-score, which was calculated in accordance with the radiomics signature. The radiomics-clinical model was based on the Rad-score and Edmondson grade. The radiomics-radiological model was established using the Rad-score and tumor size, and the combined model was established by combining the Rad-score with the Edmondson grade and tumor size.

**Table 4:** Predictive performance of six models in the training and test sets.

Models	Variables	Training set (n = 91)				Test set (n = 41)			
		AUC (95% CI)	Accuracy	Sensitivity	Specificity	AUC (95% CI)	Accuracy	Sensitivity	Specificity
Clinical	Eg	0.696 (0.591 - 0.788)	0.703	0.477	0.915	0.701(0.538 - 0.834)	0.707	0.45	0.952
Radiological	size	0.789 (0.691 - 0.868)	0.791	0.727	0.851	0.780(0.623 - 0.894)	0.78	0.75	0.81
Radiomics	Rad	0.870 (0.783 - 0.931)	0.769	0.773	0.766	0.890(0.753 - 0.966)	0.805	0.8	0.81
Radiomics-Clinical	Rad+Eg	0.881 (0.796 - 0.939)	0.813	0.795	0.83	0.933(0.810 - 0.987)	0.78	0.8	0.762
Radiomics-Radiological	Rad+ size	0.891 (0.808 - 0.947)	0.835	0.795	0.872	0.900(0.766 - 0.971)	0.805	0.8	0.81
Combined	Rad+Eg+ size	0.907 (0.828 - 0.958)	0.857	0.909	0.809	0.948(0.830 - 0.993)	0.854	0.85	0.857

Eg, Edmondson grade; Rad, Rad-score, size, tumor size; AUC, area under the receiver operating characteristic curve; CI, confidence interval.

**Figure 5:**
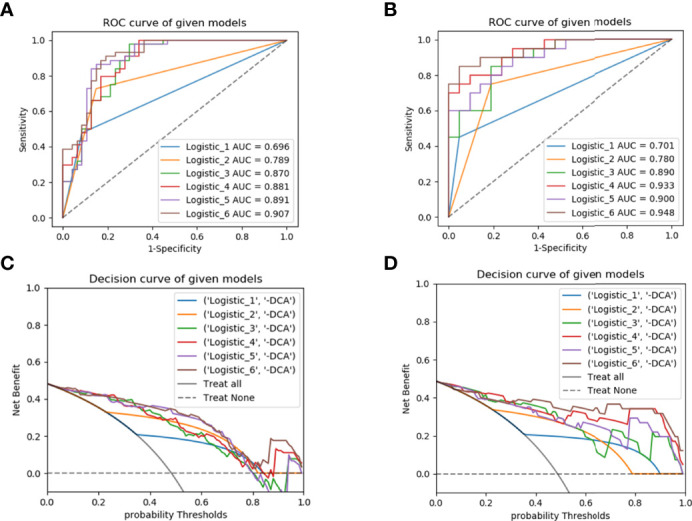
ROC curves for predicting early recurrence with different models in the training **(A)** and test **(B)** sets; the decision curve analysis (DCA) in the training **(C)** and test **(D)** sets. ROC, receiver operating characteristic; Logistic_1, clinical model; Logistic_2, radiological model; Logistic_3, radiomics model; Logistic_4, radiomics-clinical model; Logistic_5, radiomics-radiological model; Logistic_6, combined model.

The performance of the six models was evaluated in the training set and validated in the test set using ROC curves, DCA and heat maps (**Figures 5A–D**, **Supplementary Figure S4**). The performance metrics, including the AUCs, accuracy, sensitivity, and specificity were listed in **Table 4**. Notably, the combined model achieved an AUC value of 0.907 (95% CI, 0.828-0.958) (accuracy, 0.857; sensitivity, 0.909; specificity, 0.809) in the training set, which was the highest value among the prediction models. Furthermore, the similarly excellent prediction performance of the combined model in the test set was illustrated by the AUC, accuracy, sensitivity, and specificity values (**Table 4**). The radiomics model performed well in the training set (AUC, 0.87) and test set (AUC, 0.89). The DeLong test showed that the predictive performances of the radiomics-clinical and radiomics-radiological models were significantly improved compared to the models without radiomics features [AUCs (training set): 0.881, 0.891; AUCs (test set): 0.933, 0.9] (*p*<0.05), indicating that these features greatly improved the predictive performance of the traditional models. In contrast, the prediction performances of the clinical and radiological models were inferior to those of the other four models, respectively yielding AUCs of 0.696 and 0.789 in the training set and 0.701 and 0.78 in the test set.

### Development and Verification of Nomogram Based on the Combined Model

**Figure 6:**
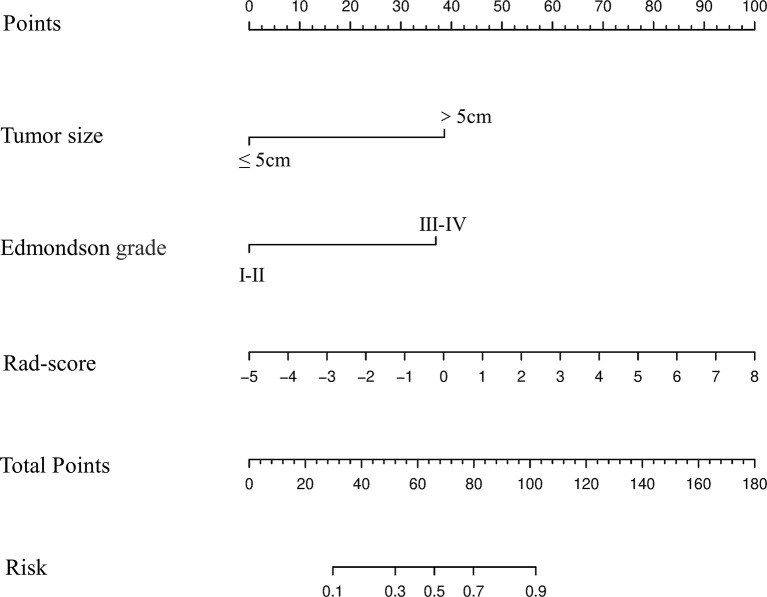
Development and performance of the nomogram based on the combined model in predicting the risk of ER in HCC patients after partial liver resection.

**Figure 7:**
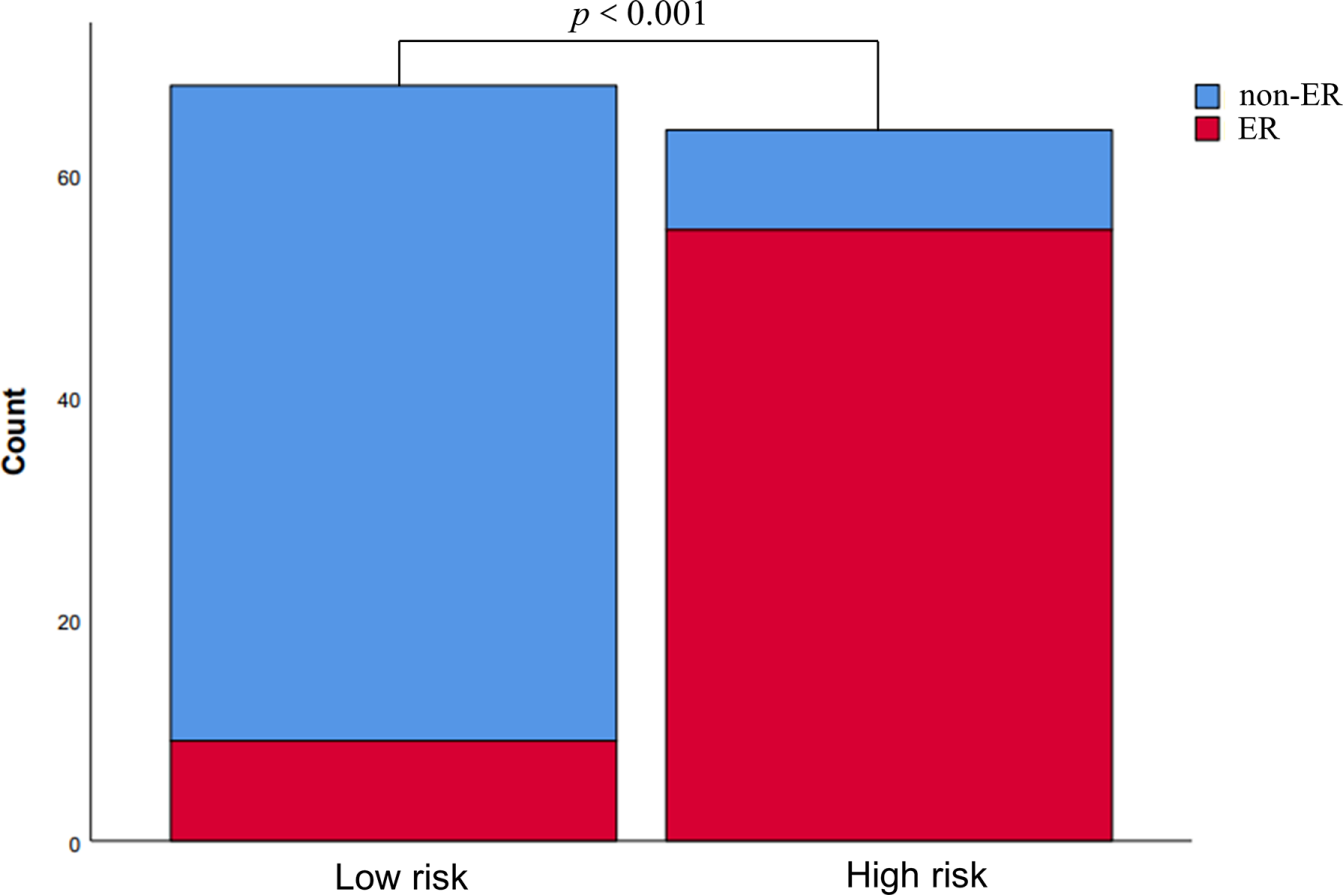
Risk stratification according to the nomogram. The risk of early recurrence was substantially higher in the high-risk group than in the low-risk group.

Based upon the combined model, we developed an intuitive, simply-to-use nomogram to predict the individual risk of ER (**Figure 6**). The optimal cut-off value was set at a risk of 0.47676 based on the Youden index. Accordingly, HCC patients were stratified into a high-risk group (risk > 0.47676) and a low-risk group (risk < 0.47676). As shown in **Figure 7**, there was a significant difference in the number of HCC patients who were predicted to develop ER between the high- and low-risk groups (*p*<0.001). Further, a significant difference in RFS was observed between the high- and low-risk groups, suggesting good clinical applicability of the nomogram in predicting ER among HCC patients.

**Figure 8:**
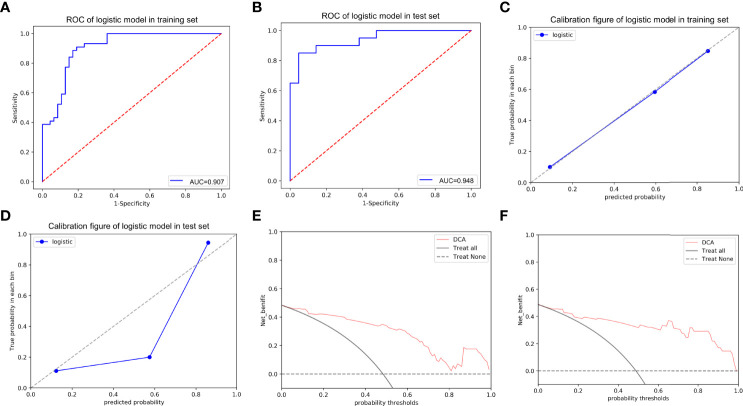
The ROC curves of the nomogram in the training **(A)** and test **(B)** sets; the calibration curves of the nomogram for predicting early recurrence in the training **(C)** and test **(D)** sets, which demonstrated good agreement with the ideal curve. The prediction performance improved as the solid line approached the dotted line. The decision curve analysis (DCA) of the nomogram in the training **(E)** and test **(F)** sets. The net benefit of the nomogram was higher compared to the treat-all-patients scenario (gray line) and treat-no-patients scenario (horizontal dotted line).

**Figure 9:**
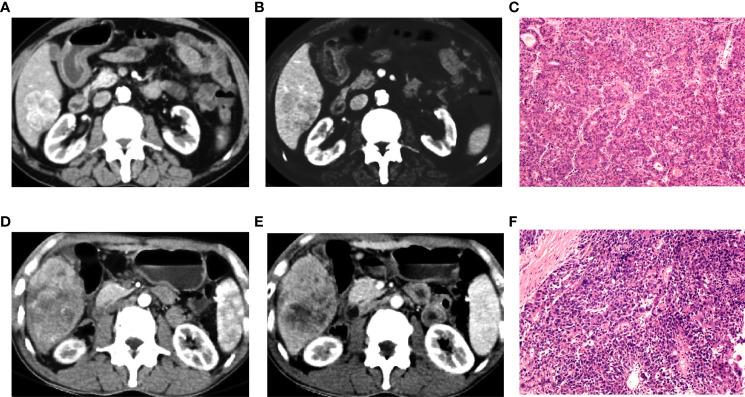
Preoperative contra-enhanced CT images and pathological pictures of two cases with hepatocellular carcinoma (HCC). **(A–C)** A 72-year-old man with non-early recurrence of HCC, the nomogram judged as low risk. **(D–F)** A 54-year-old man with early recurrence of HCC, the nomogram judged as high risk.

More notably, the AUCs of the training and test sets were 0.907 and 0.948, respectively (**Figures 8A, B**), indicating outstanding discriminative ability to predict ER and non-ER. The *p* values of the Hosmer-Lemeshow goodness-to-fit test were 0.643 for the training set and 0.962 for the test set (both *p*>0.05), indicating that the prediction of ER risk according to the nomogram was in good agreement with the actual ER status (**Figures 8C, D**). Moreover, the DCA for the training and test sets revealed good net benefits of the nomogram in clinical decisions (**Figures 8E, F**). In addition, **Figure 9** showed the nomogram had a high accuracy in predicting ER of HCC.

## Discussion

In this study of HCC patients with and without ER after partial hepatectomy, we developed and validated several CT-based radiomics models. We also created an intuitive, simple-to-use nomogram for individualized prediction of ER after comprehensive comparison and evaluation of the predictive value of CT-based radiomics of arterial and portal phases for ER in HCC patients when used individually or in combination. The nomogram had outstanding performance for ER prediction and risk stratification, as illustrated by multiple lines of evidence (1): The combined model based on the Rad-score, Edmondson grade, and tumor size had the greatest AUCs (0.907/0.948, training/test set) among six models (2); Calibration curve analysis revealed good agreement between the predicted ER using the nomogram and the actual observations (3); DCA indicated good net benefits from applying the nomogram in clinical decisions.

Recently, radiomics studies have reported that the application of standardization preprocessing of medical images can significantly reduce differences in different scanners and CT parameters (13, 16, 30). In the present study, we used several CT scanning parameters. Grey discretization and resampling were processed before feature extraction to reduce the dependence on image specification differences. We used the three-dimensional tumor volume to extract radiomics features, which can better reflect the internal heterogeneity of tumors and provide better morphological information than two-dimensional features (31, 32). Compared with a previous study by Zhu et al. (27) in which radiomics features were extracted from CT images for only the portal phase, our CT-based radiomics models had better predictive performance, as evidenced by higher AUCs. The AUCs for the radiomics model were 0.870 in our study vs. 0.749 in the previous study (training set) and 0.890 vs. 0.759 (test set), while the AUCs for the combined model were 0.907 vs. 0.800 (training set) and 0.948 vs. 0.785 (test set). We propose that the improved predictive performance observed in this study can be attributed to the valuable radiomics features of both the arterial and portal phase images extracted.

In the present study, the radiomics model was developed based on five radiomics features, specifically four GLCM features (GLCM Entropy, GLCM Energy, Cluster Prominence, Haralick Correlation) and one histogram (Voxel Value Sum) feature. These features described the relationship between adjacent pixels and the distribution of the image voxel intensity and reflected the heterogeneity and biological properties of tumors (33). The Rad-score composed of these five features was significantly higher for the ER group than for the non-ER group. This result demonstrated that the higher the radiomics feature value was, the greater the internal pixel difference of the image may be, and this difference reflected the potential heterogeneity within the tumor. In other words, a higher radiomics signature value (Rad-score) and greater tumor heterogeneity were associated with a higher risk of tumor recurrence (12). ER was related to the aggressiveness of the tumor and better reflected the heterogeneity within the tumor, which was precisely reflected by the Rad-score.

ER-associated risk factors mainly included high pathological grade, local invasion, and intrahepatic metastasis (34). A high tumor pathological grade was a dangerous risk factor for ER in HCC patients (35). In our study, the pathological grade of the tumor was also an independent factor in predicting early postoperative recurrence, which was consistent with previous studies (36, 37). Our study showed that HCC patients with higher pathological grades were more likely to develop ER after surgery, and this could be attributed to the significantly increased risk of tumor spread and intrahepatic metastasis with higher pathological grades (36). Numerous previous studies (36, 38–40) have shown that larger tumor size is associated with a higher risk of HCC recurrence, especially for tumor diameters greater than 5 cm. In agreement with these previous studies, we found that tumor size (>5 cm) was an independent factor of ER. The clinical and radiological models only showed fair discriminative performance (AUC: training set: 0.696, 0.789; test set: 0.701, 0.780). Notably, after integrating radiomics features into the clinical and radiological models, the discriminative performances improved significantly (AUC: training set: 0.881, 0.891; test set: 0.933, 0.9) (*p*<0.05), suggesting that radiomics can improve the ability to predict ER. It may merit attention that the combined model showed excellent discriminative ability, evidenced by the highest AUC values among the models in this study (AUC: training set: 0.907, test set: 0.948). Moreover, the nomogram based on the combined model was reliable for stratification of the risk of ER in HCC patients.

Our study has some limitations. Firstly, as a retrospective study, there may be selection bias. A prospective study will be needed to verify the findings. Secondly, this was a single-center study, for which the performance of the nomogram will require external validation. Thirdly, the enrolled patients had hepatitis B virus-associated HCC, and the findings may not represent patients with other etiologies and different clinicopathological features. Fourthly, the patients were classified into low- and high-risk groups in this study without actual survival analyses. However, model predictions of endpoints in dichotomous studies have also been used in previous studies (41, 42). It would be better to use more accurate survival data in future studies.

In conclusion, we established a radiomics model using robust radiomics features, and the model had good predictive performance. Integrating radiomics features into clinical and radiological models immensely improved the predictive performance of both types of models. More notably, the model constructed with a combination of radiomics features, radiological parameters, and clinicopathological characteristics showed outstanding predictive power. Our findings demonstrate that the intuitive nomogram based on the combined model is capable of predicting ER in postoperative HCC patients accurately and effectively. Therefore, the nomogram holds promise as a powerful non-invasive and personalized risk stratification tool for predicting ER in HCC patients.

## Data Availability Statement

The original contributions presented in the study are included in the article/**Supplementary Material**. Further inquiries can be directed to the corresponding author.

## Ethics Statement

The studies involving human participants were reviewed and approved by Medical Ethics Committee of Zhejiang Provincial People’s Hospital, Affiliated People’s Hospital, Hangzhou Medical College. Written informed consent for participation was not required for this study in accordance with the national legislation and the institutional requirements.

## Author Contributions

CW analyzed data, selected study, wrote the preliminary draft, reviewed of final draft. SY was responsible for conception and design, study selection, data extraction. YZ collected data and conducted radiomics analysis. LZ and SC performed study selection and statistical analysis. YL proposed the concept and design of the study. All authors contributed to the article and approved the submitted version.

## Funding

This study was supported by the Medical Science and Technology Project of Zhejiang Province (No. 2020KY019), Medical Science and Technology Project of Zhejiang Province (No. 2022KY055) and Medical Science and Technology Project of Zhejiang Province (No. 2021PY036).

## Conflict of Interest

The authors declare that the research was conducted in the absence of any commercial or financial relationships that could be construed as a potential conflict of interest.

The reviewer ZD declared a shared parent affiliation with the authors to the handling editor at the time of review.

## Publisher’s Note

All claims expressed in this article are solely those of the authors and do not necessarily represent those of their affiliated organizations, or those of the publisher, the editors and the reviewers. Any product that may be evaluated in this article, or claim that may be made by its manufacturer, is not guaranteed or endorsed by the publisher.
