# Supplementary material for: CT-Based Radiomics Nomogram Improves Risk Stratification and Prediction of Early Recurrence in Hepatocellular Carcinoma After Partial Hepatectomy
Source: Front Oncol. 2022 Jul 7;12:896002. doi: 10.3389/fonc.2022.896002 (PMC9302642; doi:10.3389/fonc.2022.896002)
Supplement: Supplementary file 1 [file DataSheet_1.docx]

**Supplementary Material**

1. **Supplementary Methods**

**1. Supplementary criteria for inclusion and exclusion**

A total of 374 hepatocellular carcinoma (HCC) patients who underwent preoperative contrast-enhanced computed tomography (CT) during the period spanning nearly 7 years from May 2013 to March 2020 in our hospital were screened to determine their eligibility. Inclusion criteria of this study were: (1) age ≥18 years, regardless of sex; (2) pathology confirmed as HCC with clear pathological grade; (3) enhanced CT examination was performed within 1 month before surgery; (4) no previous history of tumor treatment. Exclusion criteria were: (1) poor image quality (n =5); (2) Incomplete clinicopathological data or incomplete sequence in Picture Archiving and Communication System (PACS) (n=34); (3) history of antineoplastic therapy before enhanced CT examination (n=66); (4) patients with large vascular invasion or distant metastases (n=57); (5) Follow-up was less than 1 year (n=47); (6) CT imaging exceeded one month before surgery (n=33). In the end, a total of 132 subjects met the inclusion criteria according to the time of CT examination.

**2. Supplementary** **demographic, clinical and radiological data, enhanced CT images acquisition**

The demographic and clinical features of each patient were collected, including age, sex, serum hepatitis B surface antigen (HBs-Ag), serum alpha-fetoprotein (AFP), alanine aminotransferase (ALT), aspartate aminotransferase (AST), Child-Pugh grade, Barcelona Clinical Liver Cancer (BCLC) Stage, microvascular invasion (MVI), Edmondson grade. According to Edmondson grade, HCC was divided into grades I-II and III-IV. According to the threshold value, AFP, ALT, AST, and other laboratory indicators were divided into categorical variables, AFP was converted to Ln function and calculated. CT image features included tumor number, tumor size (maximum diameter), liver cirrhosis, tumor capsule, tumor margin, arterial phase (AP) peritumoral enhancement, and intratumoral necrosis. The largest lesion was selected for analysis that patients had multiple lesions. Two experienced radiologists evaluated separately, who were unaware of clinicopathological information. When two radiologists disagreed, they went over all the images and worked together until an agreement was reached.

All patients underwent abdominal unenhanced CT scans and enhanced CT scans included the AP and portal vein phase (PP). Enhanced CT images acquisition: patients fasted for six hours before scanning, 1.5ml/kg of the contrast agent Ultravist 370 was intravenously injected at a rate of 3.0-3.5ml/s. After injection of contrast agent, the AP and PP were scanned 35s and 70s, then, the images were uploaded to the PACS.

**3. Supplementary details of dimension reduction**

A total of 792 radiomics features were performed in each patient in the arterial and portal phase CT images, and the radiomics features with an intraclass correlation coefficient (ICC) of >0.80 were repeatedly tested between inter- and intra-observer, excluding radiomic features with a correlation coefficient of less than 0.80, retaining a total of 636 features. First, the variance of each feature is calculated, the features with a variance of 0 or less are preferentially excluded, the features greater than the threshold of 1 are retained, and 347 features are obtained. Secondly, 40 features were obtained using correlation analysis. These features are then dimensionally reduced using the least absolute shrinkage and selection operator (LASSO) method. LASSO is a powerful regression analysis algorithm with high-dimensional predictions. The LASSO algorithm narrows some coefficients through an absolute constraint and makes the others precisely 0. LASSO selected 17 non-zero features. Detailed information on these 17 features is shown in supplementary Table S2. To further select more valuable features, the Gradient Boosting Decision Tree (GBDT) algorithm is used to further reduce the dimensionality, and the GBDT algorithm uses the decision tree as the learning object. Using the loss function to detect residuals is a combination of gradient enhancement and decision trees. The result is the 5 most valuable radiomics features.

GBDT is comprised of the following four steps:

1. Given the initial training data, the first base learner was trained.

2. Adjust the sample according to the performance of the base learner, and more attention would be put on the sample wrongly performed by the previous learner.

3. Use the adjusted sample, train the next base learner.

4. Repeat the above process T times, combining the weighted T learners. The training process of GBDT is shown in Figure S1, and its mathematical equation is as follows:

$GBDT=\max_{s}\left[ \frac{1}{\left| S \right|}\sum_{f_{i}\in S} I(f_{i};c)-\frac{1}{\left| S \right|^{2}}\sum_{f_{i}f_{j}\in S} I(f_{i};f_{j}) \right]$

**4. The alculation formula for Rad-score and the combined model score= Constant + coefficient *features**

Rad-score = -0.0113-1.1279* GLCMEntropy_AllDirection_offset1_SD

+1.0907* VoxelValueSum

+0.7774* ClusterProminence_AllDirection_offset1_SD

-1.2881* GLCMEnergy_angle135_offset7

-0.3916* HaralickCorrelation_AllDirection_offset1_SD

Combined model score=-2.198+0.544*Rad-score+1.889* Edmondson grade+1.943* tumor size

1. **Supplementary Figures and Table**

**Supplementary Figure S1** The details of all radiomics features.

(Note. GLCM, gray-level cooccurrence matrix; GLSZM, gray-level size zone matrix; RLM, run-length matrix.)

**Supplementary Figure S2** The correlation coefficients of the features after dimensionality reduction in the training set **(A)** and the test set **(B).**

**Supplementary Figure S3** Score diagrams of the radiomics signature in the training set **(A)** and test set **(B)**. Red represents non-ER set and blue represents ER set. A score greater than 0 indicates ER set, and a score less than 0 indicates non-ER set.

**Supplementary Figure S4** Heat maps for the *p*-values of the compared AUCs in the training **(A)** and test **(B)** sets. AUC, area under the receiver operating characteristic curve; Logistic_1, clinical model; Logistic_2, radiological model; Logistic_3, radiomics model; Logistic_4, radiomics-clinical model; Logistic_5, radiomics-radiological model; Logistic_6, combined model.

**Supplementary Table S1** Detailed parameters of all the CT images

**Supplementary Table S2** Classification of radiomics features remaining after LASSO dimensionality reduction

**Supplementary Table S3** Classification of the final selected radiomics features
