# Supplementary figures and images for: CT-Based Radiomics Nomogram Improves Risk Stratification and Prediction of Early Recurrence in Hepatocellular Carcinoma After Partial Hepatectomy
Source: Front Oncol. 2022 Jul 7;12:896002. doi: 10.3389/fonc.2022.896002 (PMC9302642; doi:10.3389/fonc.2022.896002)

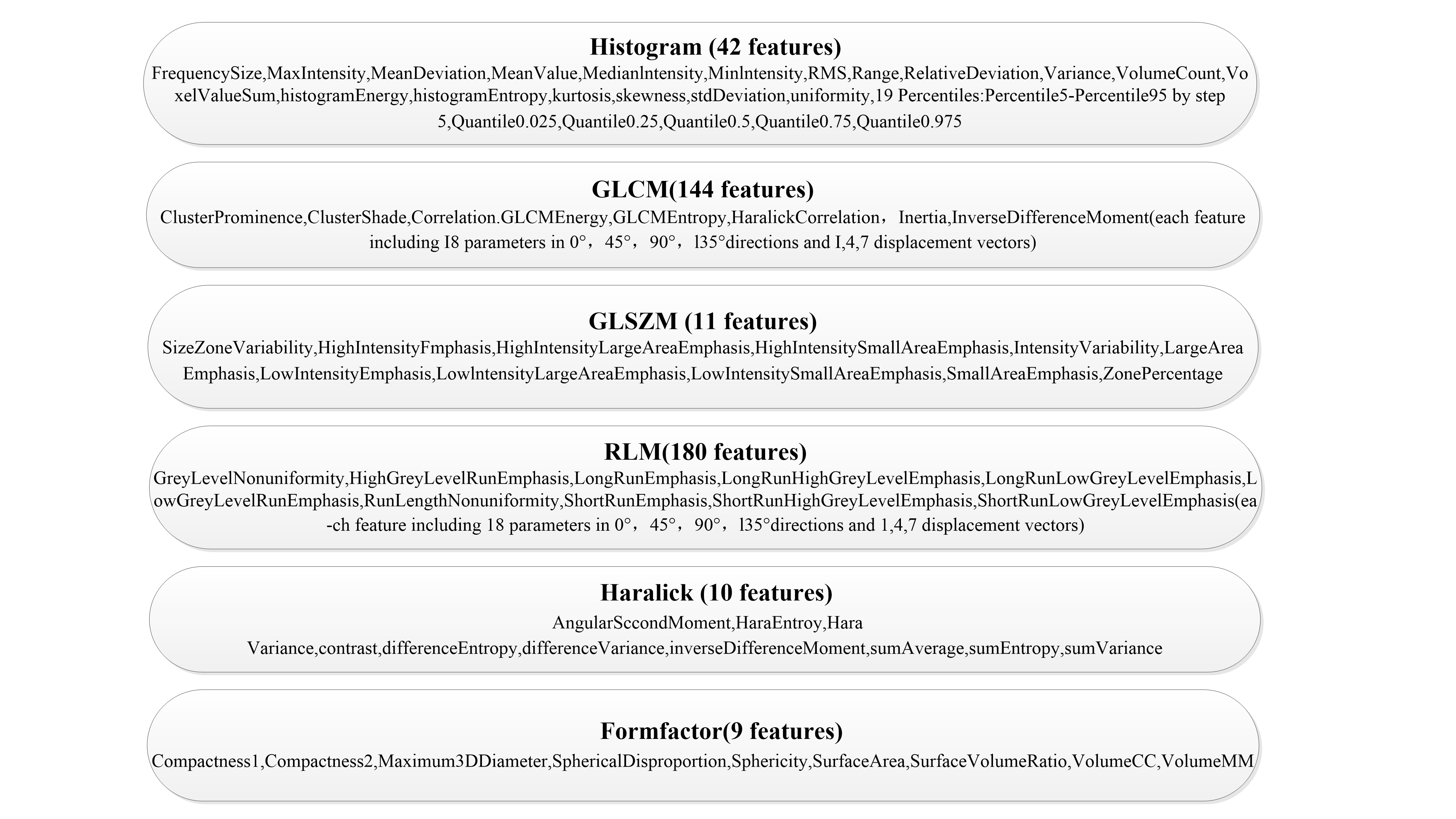

Supplement: Supplementary file 2 [file Image_1.tif]

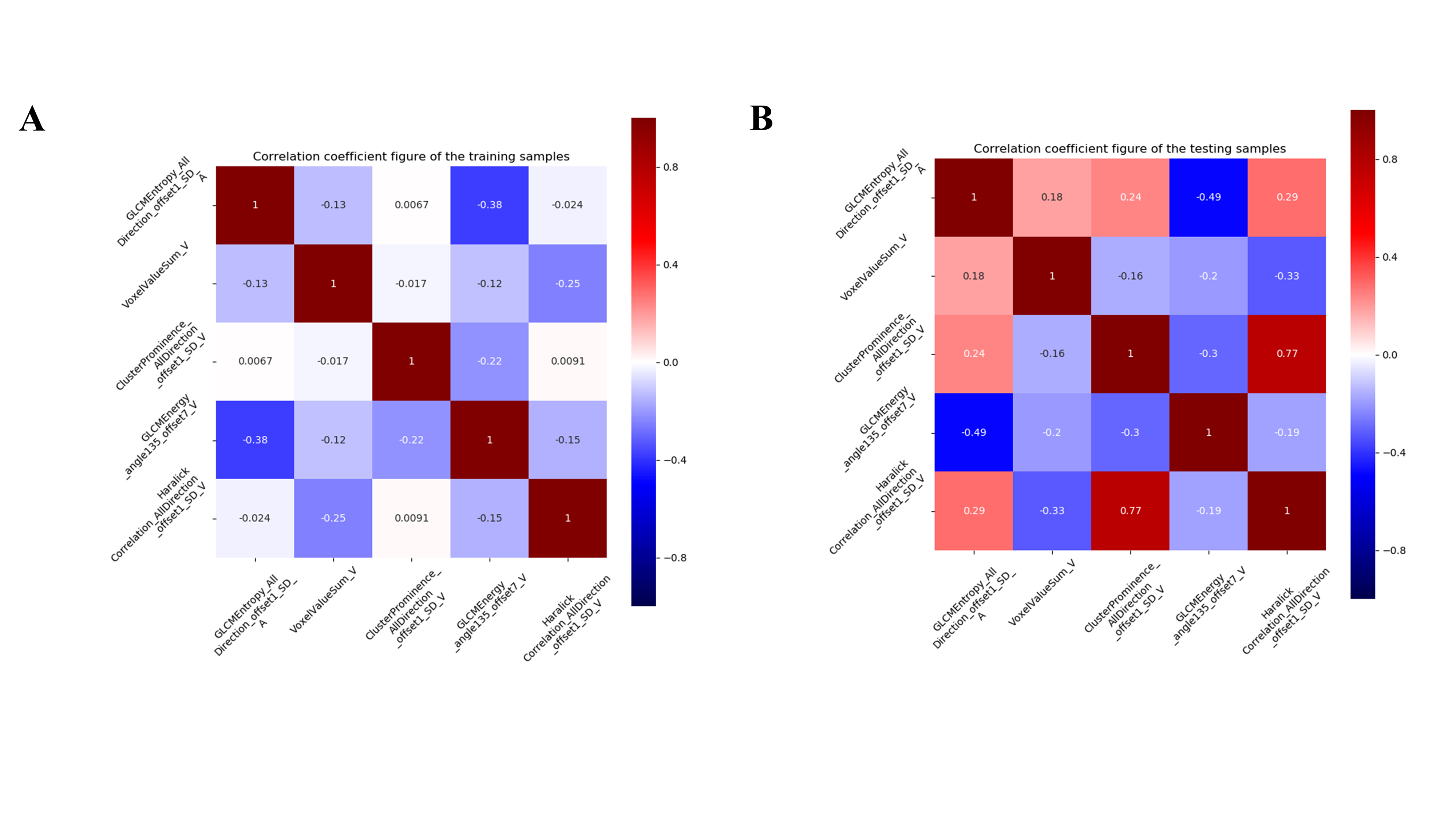

Supplement: Supplementary file 3 [file Image_2.tif]

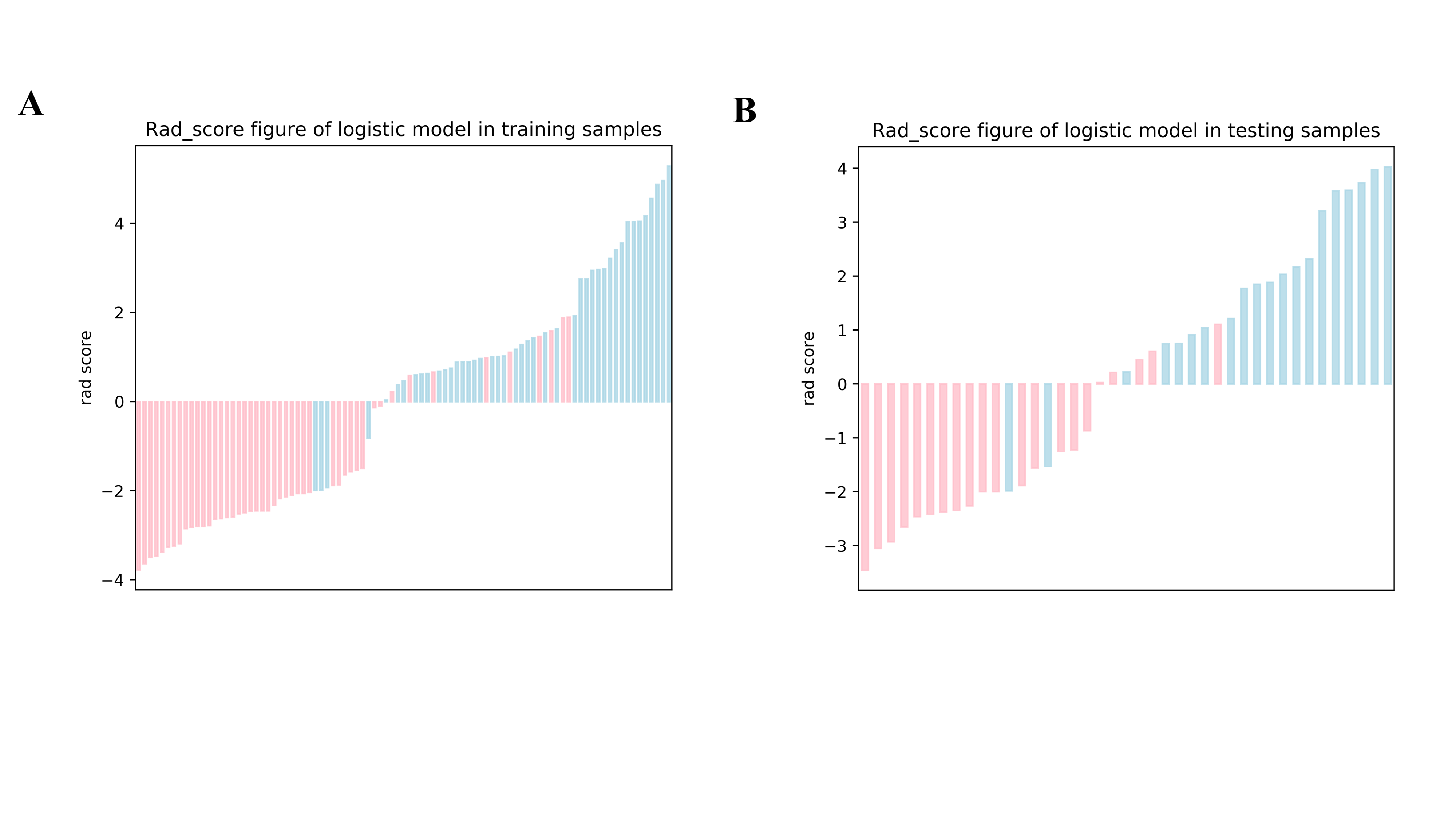

Supplement: Supplementary file 4 [file Image_3.tif]

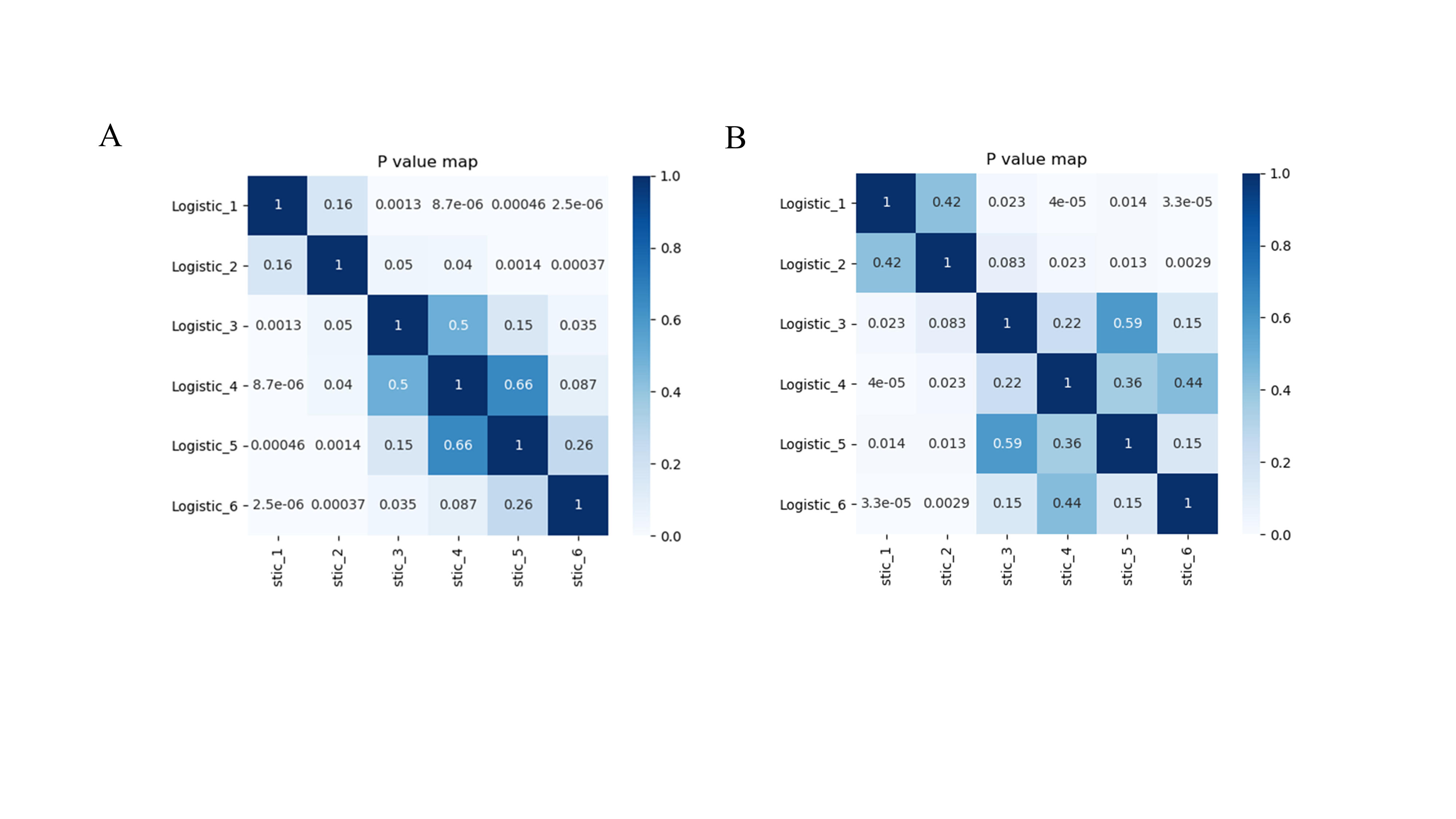

Supplement: Supplementary file 5 [file Image_4.tif]
