# Supplementary material for: CT-Based Radiomics Nomogram Improves Risk Stratification and Prediction of Early Recurrence in Hepatocellular Carcinoma After Partial Hepatectomy
Source: Front Oncol. 2022 Jul 7;12:896002. doi: 10.3389/fonc.2022.896002 (PMC9302642; doi:10.3389/fonc.2022.896002)
Supplement: Supplementary file 7 [file Table_2.docx]

| **Supplementary Table S2.** Classification of radiomics features remaining after LASSO dimensionality reduction | | |
| --- | --- | --- |
| Sequences | Features | Category |
| AP(n=6) | Correlation_angle0_offset7 | GLCM |
|  | Correlation_angle135_offset4 | GLCM |
|  | GLCMEntropy_AllDirection_offset1_SD | GLCM |
|  | LowGreyLevelRunEmphasis_AllDirection_offset1 | RLM |
|  | ShortRunEmphasis_AllDirection_offset1_SD | RLM |
|  | LowIntensityLargeAreaEmphasis | GLSZM |
| PP(n=11) | VoxelValueSum | Histogram |
|  | ClusterProminence_AllDirection_offset1_SD | GLCM |
|  | ClusterShade_angle0_offset7 | GLCM |
|  | Correlation_AllDirection_offset1_SD | GLCM |
|  | Correlation_angle135_offset7 | GLCM |
|  | Correlation_angle45_offset7 | GLCM |
|  | GLCMEnergy_angle135_offset7 | GLCM |
|  | HaralickCorrelation_AllDirection_offset1_SD | GLCM |
|  | GreyLevelNonuniformity_AllDirection_offset1_SD | RLM |
|  | HighGreyLevelRunEmphasis_angle135_offset1 | RLM |
|  | LongRunEmphasis_AllDirection_offset4_SD | RLM |
| Note. LASSO, the least absolute shrinkage and selection operator; AP, arterial phase; PP, portal vein phase; GLCM, gray-level cooccurrence matrix; GLSZM, gray-level size zone matrix; RLM, run-length matrix. | | |
