# Supplementary material for: CT-Based Radiomics Nomogram Improves Risk Stratification and Prediction of Early Recurrence in Hepatocellular Carcinoma After Partial Hepatectomy
Source: Front Oncol. 2022 Jul 7;12:896002. doi: 10.3389/fonc.2022.896002 (PMC9302642; doi:10.3389/fonc.2022.896002)
Supplement: Supplementary file 8 [file Table_3.docx]

| **Supplementary Table S3.** Classification of the final selected radiomics features | | |
| --- | --- | --- |
| Sequences | Features | Category |
| AP(n=1) | GLCMEntropy_AllDirection_offset1_SD | GLCM |
| PP(n=4) | VoxelValueSum | Histogram |
|  | ClusterProminence_AllDirection_offset1_SD | GLCM |
|  | GLCMEnergy_angle135_offset7 | GLCM |
|  | HaralickCorrelation_AllDirection_offset1_SD | GLCM |
| Note. AP, arterial phase; PP, portal vein phase; GLCM, gray-level cooccurrence matrix. | | |
